# Disorder-Specific Predictive Classification of Adolescents with Attention Deficit Hyperactivity Disorder (ADHD) Relative to Autism Using Structural Magnetic Resonance Imaging

**DOI:** 10.1371/journal.pone.0063660

**Published:** 2013-05-16

**Authors:** Lena Lim, Andre Marquand, Ana A. Cubillo, Anna B. Smith, Kaylita Chantiluke, Andrew Simmons, Mitul Mehta, Katya Rubia

**Affiliations:** 1 Department of Child and Adolescent Psychiatry, Institute of Psychiatry, King’s College London, London, United Kingdom; 2 Department of Psychological Medicine, Yong Loo Lin School of Medicine, National University of Singapore, Singapore, Singapore; 3 Department of Neuroimaging, Institute of Psychiatry, King’s College London, London, United Kingdom; 4 NIHR Biomedical Research Centre at South London and Maudsley Foundation NHS Trust and King’s College London, Institute of Psychiatry, London, United Kingdom; Bellvitge Biomedical Research Institute-IDIBELL, Spain

## Abstract

**Objective:**

Attention Deficit Hyperactivity Disorder (ADHD) is a neurodevelopmental disorder, but diagnosed by subjective clinical and rating measures. The study’s aim was to apply Gaussian process classification (GPC) to grey matter (GM) volumetric data, to assess whether individual ADHD adolescents can be accurately differentiated from healthy controls based on objective, brain structure measures and whether this is disorder-specific relative to autism spectrum disorder (ASD).

**Method:**

Twenty-nine adolescent ADHD boys and 29 age-matched healthy and 19 boys with ASD were scanned. GPC was applied to make disorder-specific predictions of ADHD diagnostic status based on individual brain structure patterns. In addition, voxel-based morphometry (VBM) analysis tested for traditional univariate group level differences in GM.

**Results:**

The pattern of GM correctly classified 75.9% of patients and 82.8% of controls, achieving an overall classification accuracy of 79.3%. Furthermore, classification was disorder-specific relative to ASD. The discriminating GM patterns showed higher classification weights for ADHD in earlier developing ventrolateral/premotor fronto-temporo-limbic and stronger classification weights for healthy controls in later developing dorsolateral fronto-striato-parieto-cerebellar networks. Several regions were also decreased in GM in ADHD relative to healthy controls in the univariate VBM analysis, suggesting they are GM deficit areas.

**Conclusions:**

The study provides evidence that pattern recognition analysis can provide significant individual diagnostic classification of ADHD patients and healthy controls based on distributed GM patterns with 79.3% accuracy and that this is disorder-specific relative to ASD. Findings are a promising first step towards finding an objective differential diagnostic tool based on brain imaging measures to aid with the subjective clinical diagnosis of ADHD.

## Introduction

Attention Deficit Hyperactivity Disorder (ADHD) is the most commonly diagnosed childhood disorder, defined by age-inappropriate problems with inattention, impulsivity and hyperactivity [1]. ADHD is a multi-systemic neurodevelopmental disorder that has consistently been associated with abnormalities in structure, function and functional and structural connectivity in fronto-striatal, temporo-parietal and fronto-cerebellar networks [2]–[11].

Despite these neurobiological underpinnings, accurate diagnosis for ADHD is a challenge and based solely on subjective clinical and rating measures, which are often unreliable with diagnostic variability between clinicians, cultures and countries [12]. It is therefore highly desirable to find objective, neuroimaging based diagnostic biomarkers to aid traditional diagnostic methods for ADHD. Attempts to find objective neuroimaging biomarkers for individual patients with ADHD, however, have been limited by the use of univariate group statistics with little success to provide individual diagnosis.

Recent multivariate pattern classification or regression analysis (MVPA) methods for imaging data, however, take into account interactions between regions (i.e. brain structure/function *patterns*) and are ideally suited to make predictions for individual subjects based on brain imaging patterns, as opposed to group-level inferences. These methods can provide sensitive and specific diagnostic indicators for individual patients with other psychiatric disorders such as autism, depression and schizophrenia [13], [14]. Gaussian Process Classifiers (GPCs) are kernel classifiers, similar to support vector machines (SVMs), which have excellent performance for MRI [15], [16] and provide probabilistic predictions that quantify predictive uncertainty. Given that MVPA take into account interrelations between regions, they are particularly suitable for multisystem disorders of widespread network abnormalities, such as ADHD [6], [7], [9]–[11].

However, to date, few imaging studies have used multivariate analysis techniques to classify ADHD patients. An early study employing discriminative features derived from resting state fMRI reported promising accuracy of 85%, but the extremely small sample (9 ADHD patients) makes the generalizability of this result uncertain [17]. Recently, a competition was launched to apply multivariate methods on a multicenter resting state functional imaging dataset of 285 children and adolescents with ADHD and 491 healthy controls, together with anatomical and phenotype data (ADHD-200 Consortium; http://fcon_1000.projects.nitrc.org/indi/adhd200/). The published studies applied a range of classification approaches including random forests, gradient boosting, multi-kernel learning and support vector machines [18]–[21]. Accuracies derived by internal cross-validation ranged from 55–78%, although the accuracies reported on an external test dataset for which diagnostic labels were withheld were substantially lower (61% for the winning team [19]). This difference was attributed to a lack of standardization between sites, leading to multiple confounds including missing data, site-specific differences in behavioral measurements, imaging acquisition, processing, and protocols, scanner quality and other unmeasured confounding and mediating variables. Furthermore, the competition dataset was highly unbalanced, with more control subjects than ADHD patients (63% and 37% respectively) and balanced accuracy measures that accommodate this imbalance [22] are consistently lower than the figures reported (e.g. 57.5% for the winning team). In addition, the competition scoring rewarded specificity more than sensitivity so that all teams reported high specificity, but poor sensitivity (21% for the winning team). Also, none of the studies used probabilistic classification models (e.g. GPC), which have important advantages for clinical studies, including accurate quantification of predictive uncertainty and the ability to adjust predictions to accommodate unbalanced diagnostic settings or variations in disease prevalence [23]. Another important issue not addressed to date is the necessity of evaluating the specificity of a classification model for ADHD relative to other disorders with overlapping symptoms and comorbidity (e.g. autism spectrum disorder (ASD)).

The aim of the present study was to address these challenges in three ways: First, to test the hypothesis that GPCs applied to grey matter (GM) images from ADHD adolescents and healthy controls acquired in the same scanner and protocol can identify distributed structural neuroimaging patterns that will provide sensitive and specific diagnostic predictors of ADHD. Second, to demonstrate that these predictors are disorder-specific to ADHD when compared to another childhood disorder, of ASD. Third, to provide patterns of predictive weights to characterize the discriminating brain regions underlying the predictions. Fourth, to carry out univariate voxel-based morphometry (VBM) case-control comparisons to investigate whether regions that accurately classify ADHD patients or controls overlap with regions that can be identified in conventional group comparisons and to replicate previous structural imaging deficit findings in frontal, temporo-parietal, striatal and cerebellar regions in ADHD.

## Materials and Methods

### Participants

Twenty-nine, mostly medication-naïve, right-handed adolescent boys with a clinical diagnosis of inattentive/hyperactive-impulsive combined ADHD between 10–18 years were recruited from clinics (Table 1). Diagnosis was assessed by an experienced child psychiatrist using the standardized Maudsley diagnostic interview [24] which contains items on symptoms of ADHD symptoms and all other major psychiatric disorders according to the DSM-IV [1]. All patients scored above clinical cut-off for hyperactive-impulsive/inattentive symptoms on the parental Strengths and Difficulties Questionnaire (SDQ) [25] and the Conners’ parent rating scale (CPRS) [26]. The majority of patients (73%) were medication-naïve, 6 patients (20%) received regular methylphenidate medication but had a washout of 48 hrs before scanning and 2 patients had been treated with methylphenidate in the past. Twenty-nine age-matched right-handed healthy boys were recruited through advertisements. They scored below clinical cut-off for the SDQ and CPRS (Table 1).

**Table 1:** Demographic and Clinical Data for Participants.

Measure	Controls (N = 29)	ADHD (N = 29)	ASD (N = 29)	*F* test	*P* value	post-hoc contrasts*
**Age (years)**	14.4 (2.48)	13.8 (1.84)	14.9 (1.86)	1.73	0.19	
**Age range**	10.7–17.9	10.5–16.5	12.1–17.9			
**IQ**	109 (10.4)	97.2 (6.91)	113 (15.7)	14.8	<0.001	ADHD<C, ASD
**IQ range**	81–125	84–109	84–138			
**CPRS ADHD score**	46.6 (6.54)	75.8 (7.29)	58.7 (7.51)	8102	<0.001	C<ASD<ADHD
**SDQ hyperactivity-impulsive/inattention** **score**	2 (1.77)	8.32 (2.09)	4.74 (1.91)	76.7	<0.001	C<ASD<ADHD
**DSM-IV total score**	45.7 (6.56)	80.1 (10.3)	58.9 (8.57)	94.9	<0.001	C<ASD<ADHD
**DSM-IV inattentive score**	45.4 (4.67)	71.8 (8.14)	55.6 (7.87)	82.6	<0.001	C<ASD<ADHD
**DSM-IV hyperactive/impulsive score**	47.4 (9.12)	83.5 (9.85)	60.4 (12.4)	72.3	<0.001	C<ASD<ADHD
**ADOS Communication scores**	–	–	2.24 (1.48)			
**ADOS Social scores**	–	–	7.35 (3.92)			
**ADOS stereotyped behaviour scores**	–	–	1.11 (0.99)			
**ADI social scores**	–	–	16.3 (4.59)			
**ADI communication scores**	–	–	13.9 (3.77)			
**ADI repetitive behaviour scores**	–	–	5.17 (2.85)			

Data expressed as mean (SD). Abbreviations: IQ = intelligence quotient as assessed with the Wechsler Abbreviated Scale of Intelligence; ADHD = Attention Deficit Hyperactivity Disorder; ASD = Autism Spectrum Disorder; CPRS = Conners’ Parent Rating Scale; SDQ = Strengths and Difficulties Questionnaire; ADOS = Autism Diagnostic Observation Schedule; ADI = Autism Diagnostic Interview. *Post-hoc t-tests were Bonferroni corrected.

Nineteen right-handed age-matched medication-naïve adolescent boys with ASD were recruited through clinics. ASD diagnosis was made using ICD-10 research diagnostic criteria [27] confirmed by the Autism Diagnostic Interview-Revised (ADI-R) [28] and the Autism Diagnostic Observation Schedule (ADOS) [29]. Boys with ASD were excluded if they scored above 7 on the hyperactive-impulsive/inattentive ratings on the SDQ. ASD boys had to score above and ADHD boys below the clinical threshold for the Social Communication Questionnaire (SCQ) [30](Table 1).

Participants were excluded if they had comorbid psychiatric disorders including learning disability, reading, speech or language disorder as assessed with the Maudsley diagnostic interview, neurological abnormalities, epilepsy, drugs or substance abuse and IQ <70 on the Wechsler Abbreviated Scale of Intelligence (WASI) [31]. Participants were paid £50 for taking part in the study. Written informed consent was obtained from participants over 16 years and from parent/guardians of the participants if they were under 16 years of age. None of the participants had compromised capacity to consent. The study was approved by the South London Research Ethics committee.

### MRI Image Acquisition

Images were acquired using a 3 T GE Signa HDx system (General Electric, Milwaukee, WI, USA) at the Centre for Neuroimaging Sciences, Institute of Psychiatry, King’s College London, UK. The body coil was used for RF transmission and an eight channel head coil for RF reception. High resolution structural 3D T1-weighted SPGR images were acquired. Full brain and skull coverage was required for each subject and detailed quality control carried out on all MR images according to previously published quality control criteria [32], [33].

### VBM-DARTEL Image Preprocessing

The images were first visually inspected for artifacts and structural abnormalities. Next, VBM [34] was conducted to investigate differences in GM volumes between the ADHD and control groups using SPM8 (Statistical Parametric Mapping, Wellcome Department of Imaging Neuroscience, London, UK, http://www.fil.ion.ucl.ac.uk/spm). The T1-weighted volumetric images were preprocessed using the VBM protocol with modulation [35] where the images were first segmented into GM, white matter (WM), and cerebrospinal fluid (CSF). The Diffeomorphic Anatomical Registration using Exponential Lie algebra (DARTEL) algorithm was applied to the segmented brain tissues to generate a study-specific template, and to achieve an accurate inter-subject registration with an improved realignment of smaller inner structures [36]. The normalized modulated segmented GM images were next affine transformed into MNI space and smoothed with an 8-mm isotropic Gaussian kernel, providing a balance between predicted subcortical and cortical effects, and to accommodate the assumptions of Gaussian Random Field Theory and the Matched Filter Theorem, and subsequently used as input into the MVPA classification algorithm. The use of an 8 mm smoothing kernel was chosen in order to provide a balance between predicted subcortical and cortical effects since effects in small subcortical structures might not be detected with larger smoothing kernels (>8 mm) optimised for putative large cortical clusters while effects in larger cortical clusters might not be detected with smaller smoothing kernels. Therefore, according to the requirements of both Gaussian Random Field Theory and Matched Filter Theorem and to be consistent with most structural MRI studies, we utilise a smoothing kernel of 8 mm across the whole brain.

### Statistical Analysis

#### Multivariate pattern recognition approach: Gaussian Process Classification

We used a binary Gaussian Process Classifier (GPC) [15], [16], which is a supervised pattern recognition approach that assigns a predictive probability of group membership to each individual based on a set of “training” data. GPCs are kernel classifiers similar to the widely used Support Vector Machines (SVMs) and have shown high levels of performance for neuroimaging data [15], [16]. Moreover, GPCs have advantages over SVMs especially for clinical applications where classes are likely to be heterogeneous. Specifically, the probabilistic predictions they provide encode a measure of predictive confidence that quantifies diagnostic uncertainty and can capture variability within clinical groups. More importantly, probabilistic methods furnish simple and effective methods to compensate for unbalanced training datasets (as outlined below) and probabilistic predictions can also be easily adjusted to compensate for the prior frequency of diagnostic classes in experimental populations. This means that inference remains coherent in classification scenarios where the frequency of each class in the test set are different from the frequencies observed in the training set and is useful to accommodate variations in disease prevalence [15], [16], [23].

We used a linear binary GPC to discriminate between ADHD and control subjects as implemented in the Pattern Recognition for Neuroimaging Toolbox (PRoNTo) software (http://www.mlnl.cs.ucl.ac.uk/pronto) (for details see [15], [16]). Whole-brain GM images were used as input patterns and the expectation propagation approximation was used to estimate the posterior predictive distribution, which produces excellent performance for binary classification. We employed a leave-one-subject-out cross-validation (LOO-CV) approach to assess classifier generalizability, whereby we excluded a matched pair of subjects to comprise the test set, and inferred all parameters from the remaining data (training set), before applying this classifier to predict the labels for the test samples. For the binary classifiers, performance was evaluated using receiver operating characteristic (ROC) curves derived from the probabilistic predictions and classification accuracy which measures the classifier performance in a categorical sense. The ROC curve compares the classifier’s true positive rate (i.e. the percentage of ADHD subjects correctly classified as ADHD) and false positive rate (i.e. the percentage of misclassified control subjects) as the decision threshold is varied. The area under the curve (AUC) is thus a summary measure of the performance of the classifier across all decision thresholds, where a classifier with perfect classification would achieve an AUC of 1 and a classifier guessing at chance-level would achieve an AUC of 0.5.

To derive categorical predictions from the probabilistic predictions derived from the GPC, we thresholded the predictions according to the frequency of classes in the training set (i.e. 0.5 if the classes are balanced). We computed the sensitivity and specificity as the proportion of ADHD patients or controls respectively having the correct label, averaged across all test splits. Finally we computed the (balanced) accuracy as the mean of sensitivity and specificity, which quantifies the overall categorical classification performance of the classifier in a way that accommodates potential class imbalance in the data.

We also employed an additional metric for the binary classifiers, known as “target information”. This measure quantifies the information gain obtained by the classifier and is measured in bits. A simple baseline classifier that always makes probabilistic predictions based on the proportion of samples from each class in the training set will obtain a target information of zero and a classifier that obtains perfect accuracy and perfect confidence will obtain a target information of one. See [16], [37], [38] for more details.

For the multi-class classifier, we primarily assessed performance using the obvious generalizations of sensitivity, positive predictive value and balanced accuracy, because the other metrics do not generalize naturally to the multi-class setting.

Permutation testing was used to derive a *p*-value to determine if the balanced accuracy exceeded chance levels (50%). This is preferred to a parametric test, as it does not require distributional assumptions. To achieve this, we permuted class labels 1000 times, each time randomly assigning patient and control labels to each image and repeating the entire cross-validation procedure. We then counted the number of times the permuted test accuracy was higher than the one obtained for the true labels. This number was then divided by 1000 to give a *p*-value for the classification.

We also correlated the GPC predictive probabilities with age, IQ, SDQ hyperactivity-impulsive/inattention subscale and the CPRS ADHD T-scores.

#### Specificity of the classifier to ADHD: classification of ADHD vs. non-ADHD individuals

To establish the degree of clinical specificity of the classification algorithm to ADHD, we wanted to assess whether the clinical specificity of the classifier remained high, when we added another disorder-group to the control group, i.e. we aimed to discriminate ADHD from “non-ADHD”, including another disorder group.

For this purpose, we included another sample of 19 patients with ASD and trained a linear binary GPC to discriminate ADHD from a combined group of ASD and healthy controls (i.e. “not ADHD”). In this case, we employed a LOO-CV approach where we excluded a single subject per cross-validation fold, and assessed classifier performance by the same metrics noted above. Note, however, that since this dataset is unbalanced, we employed a categorical decision threshold defined by the frequency of classes in the training set.

#### Specificity of the Classifier to ADHD: Classification of ADHD vs. ASD Patients

To further establish the degree of clinical specificity of the classification algorithm to ADHD relative to ASD specifically, we trained another linear binary GPC to discriminate ADHD from the group of 19 ASD patients. As above, we employed a LOO-CV approach where we excluded a single subject per cross-validation fold, and assessed classifier performance by the same metrics noted above. Since this dataset is unbalanced, we employed a categorical decision threshold defined by the frequency of classes in the training set.

#### Multi-class Gaussian Process Classification of GM tissues

In addition, we also performed a 3-class GPC classification which aims to simultaneously discriminate each group (ADHD, healthy controls and ASD) from one another. As above, we employed a LOO-CV approach where we excluded a single subject per cross-validation fold, and assessed classifier performance primarily in terms of balanced classification accuracy. In contrast to the binary classifiers described above, we use the Laplace approximation to the posterior predictive distribution (see [16], [37], [38] for details). A direct multi-class classifier of this type provides a pattern of predictive weights for each of the classes. As in the binary context, the weight vector coefficients encode the contribution of each voxel to the decision function for each group relative to the others. A high positive score in the weight vector for a given group denotes a strong positive contribution to a prediction in favor of that group, while a high negative score for the same group denotes a strong negative contribution.

#### Mass-univariate Approach: VBM Analysis

Group differences were evaluated for GM, WM, CSF volumes and total intracranial volume (TIV) (the sum of GM, WM and CSF volumes) obtained in the tissue segmentation step of the VBM-DARTEL preprocessing. The normalized modulated and smoothed GM images in each group were entered into a voxel-wise two-sample *t*-test analysis where conventional VBM-type analysis was employed using a relatively stringent significance threshold of p0.05, family-wise error rate (FWE) corrected at the cluster level. Cluster sizes were adjusted for smoothness non-uniformity by means of the VBM5 toolbox [39]. To facilitate the interpretation of the multivariate pattern findings and to further compare the results obtained with VBM and GPC, we lowered the threshold to a more lenient cluster value of *p*<0.001 uncorrected for multiple comparisons to identify regions that may have contributed to the classification but did not survive multiple comparison correction.

## Results

### Participant Characteristics

Groups did not significantly differ in age, but in IQ (see Table 1). ADHD boys had significantly lower IQ scores relative to the other two groups, which is typical in this population [40].

### Gaussian Process Classification of GM Tissues (ADHD versus Healthy Controls)

**Figure 1:**
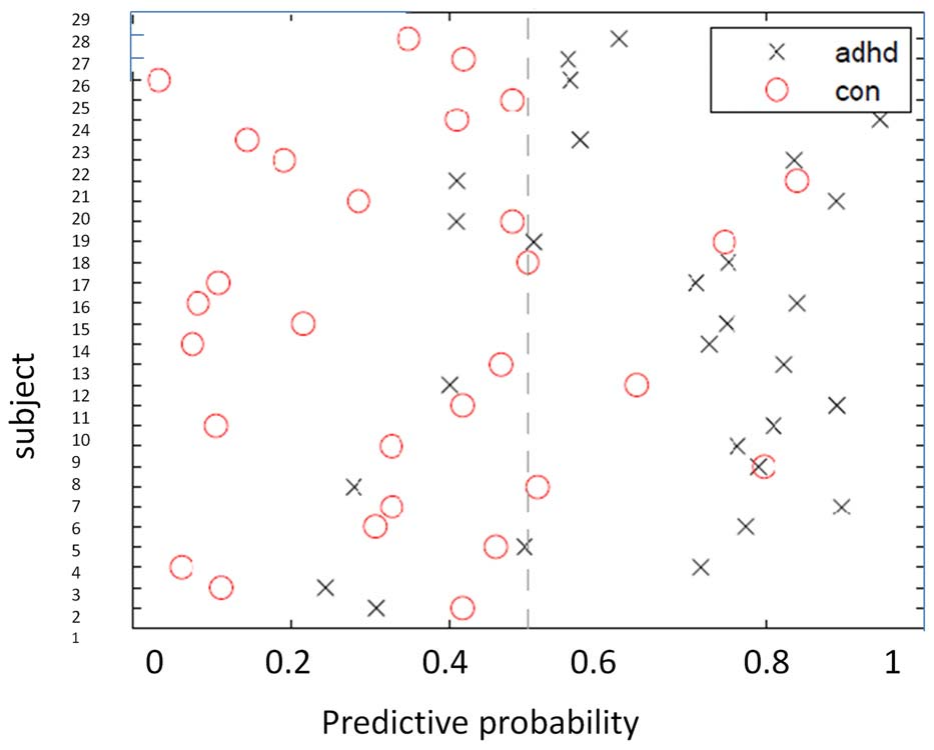
Predictive Probabilities for the Gaussian Process Classifier discriminating ADHD and Controls. The x-axis describes the probability with which each subject is predicted to be an ADHD patient (equal to 1- the probability of being a control).

GPC based on whole brain analysis differentiated ADHD patients from healthy controls with 79.3% accuracy (p<0.001). The sensitivity of classification for the ADHD group was 75.9%, while the specificity of the classification for controls was 82.8%. The positive and negative predictive values (PPV/NPV) for the classifier were 81.5% and 77.4%, respectively (Figure 1). The area under the ROC curve (i.e. AUC) was 0.83 and the classifier delivered 0.28 bits of information per test case.

**Figure 2:**
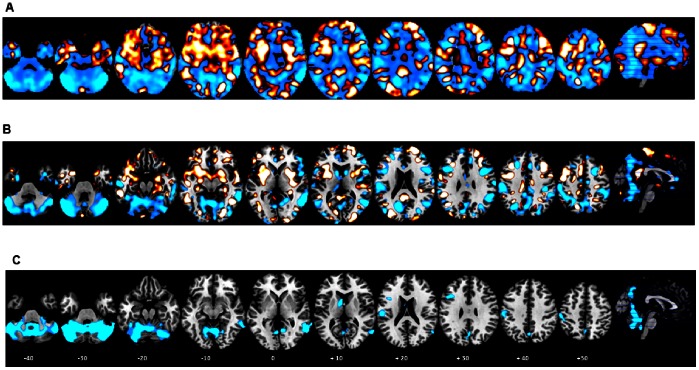
2-class Multivariate and Conventional Maps. A. Multivariate discrimination weight map for ADHD vs. Controls (unthresholded). Gaussian Process Classification classified ADHD patients and healthy controls with 82.8% and 75.9% sensitivity, respectively; leading to an overall accuracy of 79.3%. Multivariate discrimination weight-map –intensity values illustrate the relative positive weight distributions (ADHD; orange) and negative weight distributions (controls; light blue). Within each colour code, the lighter colors (i.e., light orange-yellow, light blue) indicate strongest weights for the GPC analyses and for the conventional mass-univariate case-control comparison lighter colors indicate higher p-values of structural differences. B. Multivariate discrimination weight map (thresholded). The map only shows voxels with a weight value above 40% of the maximum weight value C). Conventional mass-univariate t-statistic map. Controls had increased grey matter relative to patients, thresholded at cluster-wise p<0.001 uncorrected. No areas showed increased grey matter in ADHD relative to controls.

Figure 2A shows the discrimination weight map (w-map) showing global spatial patterns that best discriminated the groups. The weights represent spatially distributed patterns showing the relative contribution of each voxel to the decision function with positive weights indicating a positive contribution toward predicting ADHD and negative weights indicating a positive contribution toward predicting controls.

Regions in the discriminating patterns predictive of controls included predominantly bilateral hemispheres and cerebellar vermis, middle temporal, inferior and dorsolateral prefrontal cortices (IFC/DLPFC), caudate and thalamus, precuneus/cuneus and inferior and superior parietal regions as well as left ventromedial frontal cortex, including anterior cingulate (ACC) and supplementary motor area (SMA) (Figure 2A,B).

Regions in the discriminating pattern predictive of ADHD patients were most predominantly in earlier developing ventral brain regions relative to the more dorsal counterparts that classified healthy controls, such as bilateral ventrolateral, premotor and ventral temporal cortices, limbic regions including hippocampus, amygdala, hypothalamus, ventral striatum/putamen, insula, posterior cingulate and brain stem. There was also a small cluster in the inferior vermis that had greater classification weights for ADHD, as opposed to the rest of the cerebellum that showed greater weights for classifying controls (see above) (Figure 2A,B).

No correlations were observed between GPC probability measures and clinical measures within each group.

To test for potential effects of IQ or age on the classification patterns, we also correlated these within each group. No significant correlations were observed.

### Gaussian Process Classification of GM Tissues (ADHD vs. non-ADHD)

**Figure 3:**
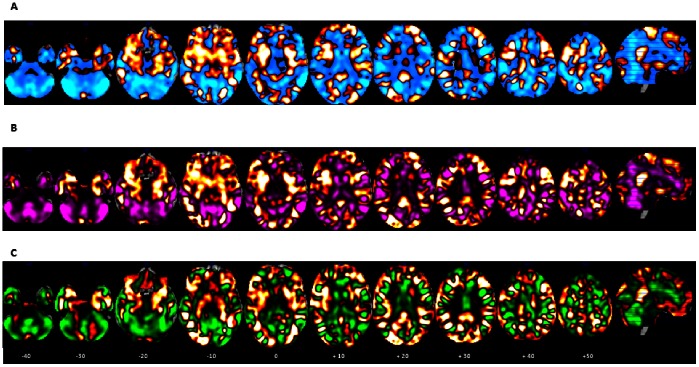
2-class multivariate weight maps. A) Multivariate discrimination weight map for ADHD vs. Controls (unthresholded). Gaussian Process Classification classified ADHD patients and healthy controls with 82.8% and 75.9% sensitivity, respectively; leading to an overall accuracy of 79.3%. Multivariate discrimination weight-map –intensity values illustrate the relative positive weight distributions (ADHD; orange) and negative weight distributions (controls; blue). Within each colour code, the lighter colors (i.e., light orange-yellow, light blue) indicate strongest weights for the GPC analyses. B) Multivariate discrimination weight map for ADHD vs. non-ADHD (unthresholded). Gaussian Process Classification classified ADHD patients and non-ADHD with 79.3% and 77.1% sensitivity, respectively; leading to an overall accuracy of 78.2%. Multivariate discrimination weight-map–intensity values illustrate the relative positive weight distributions (ADHD; orange) and negative weight distributions (non-ADHD; violet). Within each colour code, the lighter colors (i.e., light orange-yellow, light violet) indicate strongest weights for the GPC analyses. C) Multivariate discrimination weight map for ADHD vs. ASD (unthresholded). Gaussian Process Classification classified ADHD patients and ASD patients with 93.1% and 68.4% sensitivity, respectively; leading to an overall accuracy of 80.8%. Multivariate discrimination weight-map–intensity values illustrate the relative positive weight distributions (ADHD; orange) and negative weight distributions (ASD; green). Within each colour code, the lighter colors (i.e., light orange-yellow, light green) indicate strongest weights for the GPC analyses.

The binary classifier trained to discriminate ADHD patients from the group of healthy controls and ASD patients (i.e. non-ADHD) achieved a balanced accuracy of 77.1% (p<0.001). The sensitivity of this disorder-specific classification for the ADHD group was 79.3% and the specificity was 75.0%. The PPV and NPV were 65.7% and 85.7% respectively, the AUC was 0.83 and the classifier delivered 0.26 bits of information per test case. Figure 3B shows the discrimination w-map showing global spatial patterns that best discriminated the ADHD and non-ADHD groups.

No significant correlations were observed between GPC and either age, IQ or clinical measures within either group.

### Gaussian Process Classification of GM Tissues (ADHD vs. ASD)

The binary classifier trained to discriminate ADHD patients from ASD patients achieved a balanced accuracy of 85.2% (p<0.001). The sensitivity of this disorder-specific classification for the ADHD group was 86.2% and the specificity was 84.2%. The PPV and NPV were 89.3% and 80.0% respectively, the AUC was 0.91 and the classifier delivered 0.33 bits of information per test case. Figure 3C shows the discrimination w-map that shows the global spatial patterns that best discriminate the ADHD and ASD groups. The weights represent spatially distributed patterns showing the relative contribution of each voxel to the decision function with positive weights indicating a positive contribution toward predicting ADHD and negative weights indicating a positive contribution toward predicting ASD. Regions in the discriminating patterns predictive of ASD included predominately the bilateral cerebellar hemispheres and cerebellar vermis, superior and middle temporal gyri, predominantly right inferior cortex, anterior cingulate and SMA, caudate, thalamus, and limbic areas such as parts of the insula, (anterior) nucleus accumbens, (dorsal) substantia nigra and (dorsal) fornix.

No significant correlations were observed between GPC and either age, IQ or clinical measures within each group.

### Multi-class Gaussian Process Classification of GM Tissues

The 3-class classifier trained to discriminate among ADHD, healthy controls and ASD adolescents achieved a balanced accuracy of 68.2% (p<0.001) which easily exceeded the 33.33% accuracy that would be predicted for a 3-class classifier by chance. The sensitivity of this classification for the ADHD, healthy controls and ASD groups were 75.9%, 65.5%, and 63.2%, respectively; and the PPV were 62.9%, 73.1% and 75%, respectively (see Figure S1 for the 3-class w-maps).

Therefore, the GPC accurately discriminated all classes from one another. We emphasize that multi-class classification is a more difficult problem than binary classification and the accuracies listed above reflect substantially better predictive performance than would be the case for the same numerical values derived from a binary classifier.

### VBM-DARTEL Analysis of GM Volume Differences

The ADHD group had significantly smaller GM, CSF and TIV volumes than controls (Table 2). Using the conventional voxel-wise two-sample *t*-test VBM analysis with a stringent FWE correction of *p*<0.05 at cluster level, the ADHD group had significantly smaller GM volume in right cerebellum (anterior and posterior lobe) (*p*<0.0001) and in left inferior parietal lobule (*p*<0.03) (Table 3, Figure 2C). At an uncorrected cluster threshold of p<0.001, the ADHD group also had smaller GM volumes in bilateral IFC, right middle and inferior temporal gyri, and in a more ventral region of left inferior parietal lobe reaching into postcentral gyrus (Figure 2C, Table 3). Given that based on previous meta-analyses of structural MRI studies in ADHD [2], [4], we also hypothesized reduced GM volumes in the basal ganglia, we tested for trend-level findings in this region and found reduced GM volumes in left caudate (MNI coordinates (x,y,z): 8; 8,-6) in ADHD relative to healthy control boys, at a trend level of an uncorrected cluster threshold of p<0.086.

**Table 2:** Global volume group differences in ADHD and controls.

	Controls (N = 29)	ADHD (N = 29)	*t* test	*p* value
**GM volume (ml)**	790 (53.5)	749 (59.9)	2.69	0.009
**WM volume (ml)**	515 (44.0)	497 (47.3)	1.50	0.138
**CSF volume (ml)**	341 (42.9)	318 (30.2)	2.30	0.025
**TIV volume (ml)**	1646 (130)	1565 (129)	2.36	0.022

Data expressed as mean (SD). ADHD: Attention Deficit Hyperactivity Disorder;

GM: grey matter; WM: white matter; CSF: cerebrospinal fluid; TIV: total intracranial volume ( =  GM+WM+CSF volumes).

**Table 3:** Reduced grey matter in ADHD relative to healthy control boys in the traditional VBM analysis.

Brain regions	Brodmann area	Talairach Coordinates	Voxels	Cluster p-value
**R & L Cerebellum**		**27;−58; −44**	**17628**	**<0.0001**
**L inferior parietal/pre−/postcentral**	**40/2/1/3/4**	**−51; −18;45**	**765**	**0.007**
L inferior frontal	44/45/9	−40;20;31	981	0.003
R inferior frontal	44/45	48;15;19	421	0.034
L inferior parietal/postcentral	40/2/1/3	−56; −19;22	599	0.014
R middle/inferior temporal	21/37/39	60; −52;0	1255	0.001

Regions that survived a cluster-wise FWE correction at p<0.05 are indicated in bold. All other regions were observed at an uncorrected cluster-wise p<0.001. No increase in grey matter was observed for ADHD relative to controls.

## Discussion

We show that it is possible to correctly classify mostly medication-naïve ADHD boys from controls based on their GM patterns with an overall accuracy of 79.3%, providing a sensitivity of 81.5% and a specificity of 77.4%. Crucially, this classifier also demonstrated excellent disorder-specificity relative to ASD with a sensitivity of 86.2% and a specificity of 84.2%, showing that 1) the patterns of structural abnormality predictive of ADHD are not attributable to psychopathology in general, such as ASD, which shares symptoms and is frequent comorbid with ADHD [41] and 2) that all diagnostic groups could be simultaneously and accurately discriminated from one another.

The regions of the discriminative pattern most predictive of ADHD were mostly in earlier developing ventral frontal, premotor, temporal, limbic and brain stem regions. The regions of the discriminative pattern most predictive of controls included typically later developing more dorsolateral and inferior prefrontal regions, ACC/SMA, dorsal striatum, thalamus and inferior parietal areas that form fronto-striato-parieto-cerebellar networks mediating the higher level cognitive control, attention and timing functions that are impaired in ADHD[6], [7], [9]–[11]. Furthermore, several of the IFC, cerebellar and inferior parietal regions that had higher weights for classifying healthy controls based on the multivariate analyses, were also reduced in GM in ADHD relative to healthy boys, suggesting they were GM deficit regions. The findings of increasing classification weights for ADHD in earlier developing ventral fronto-temporo-limbic networks by contrast to more dorsal DLPFC and IFC striato-parieto-cerebellar networks for controls are in line with evidence from univariate longitudinal structural imaging studies of a delay in brain maturation for ADHD [42], [43].

The classification accuracy findings of over 79% based on ADHD brain structure measures and its disorder-specificity relative to ASD with a 85% of accuracy are promising and, if replicated, suggest that it may be possible in the future to use machine learning based pattern recognition analyses to aid in the differential diagnostic classification of ADHD with a more objective and reliable measure such as a short structural MRI scan.

The pattern classification analysis revealed relatively high prediction accuracy for ADHD, with a classification accuracy of 79.3%. The overall accuracy obtained in this study is thus not only higher than all studies derived from the ADHD-200 competition using resting state and structural MRI data (ranging between 67% and 76%)[18]–[21], but it also demonstrates high sensitivity and high specificity relative to healthy controls and ASD patients. Finally, our approach is readily interpretable in that it provides a discriminative pattern that quantifies the discriminative value of different brain regions and can be easily related to VBM findings that quantify difference between disease groups in a univariate manner.

Interestingly, the distributed GM patterns that showed the highest weights for classifying the ADHD boys were in predominantly subcortical areas, including most parts of the limbic system (amygdala, hippocampus, hypothalamus, insula, ventral striatum and posterior cingulate) and in brain stem, as well as in more ventral frontal, premotor and temporal regions, which contrasted with the distributed network of more dorsal DLPFC and IFC, dorsal striatum and inferior parietal areas that correctly classified controls. Furthermore, the predictive probabilities for ADHD patients were correlated with ADHD severity ratings, reinforcing their diagnostic classification utility. Subcortical as well as ventrolateral frontal and striatal regions that classified ADHD develop earlier than the more dorsal cortical and striatal brain regions that were characteristic for the classification of controls [44], [45]. The pattern classification findings are hence in line with the notion of a maturational delay of GM development in ADHD patients, as demonstrated in univariate analyses that showed a delay in ADHD in the maturation of cortical thickness and surface morphology of between 2 and 5 years, most prominently in dorsolateral prefrontal, superior temporal and inferior parietal brain regions [42], [43], [46]. This is further supported by the fact that the same lateral DLPFC/IFC-caudate-parieto-cerebellar networks that classified controls in their GM mediate the late developing higher level functions of cognitive control, timing and attention [47] that are typically impaired in ADHD patients in cognition and functional activation[6], [9]–[11], [48].

All brain regions that were reduced in ADHD patients in their GM relative to controls in the univariate analyses overlapped with brain regions that showed a higher weight for classifying controls, suggesting that the regions in the multivariate patterns predictive of controls reflect GM deficit areas in ADHD. The findings of reduced GM in the lateral parts and vermis of the cerebellum, in bilateral inferior frontal, left parietal, and right temporal cortices replicates previous findings of reduced GM in these regions in whole brain [3], [49] as well as region of interest (ROI) analyses [5], [50]. The findings of the largest and most significant reduction in GM in the cerebellum replicates previous findings [51], [52], also outlined in a meta-analysis of ROI structural MRI studies [5]. The cerebellum is one of the latest areas to develop [53], together with the frontal and superior temporal lobes [45] and the finding is hence in line with the notion that ADHD patients have deficits in late maturing brain regions, likely due to a maturational delay. We only observed reduced GM in the caudate at a more lenient uncorrected threshold of p<0.078, and in the left hemisphere, which was unexpected, given that a reduction of right basal ganglia GM was the most consistent finding in two recent meta-analyses of whole brain structural MRI studies [2], [4]. The sample size of this study is relatively small for univariate structural image analyses and the basal ganglia GM deficits may only be observable with larger sample sizes.

Importantly, we demonstrated that the achieved classification is disorder-specific to ADHD, as similar and even increased accuracy was achieved when we added a psychiatric control group of ASD adolescents and when we compared ADHD and ASD alone with each other, where we achieved a sensitivity of 86.2% and a specificity of 84.2%. The findings extend the previous literature of multivariate pattern analyses of ADHD by showing for the first time that the patterns that classify ADHD patients are disorder-specific and do not classify ASD patients. The findings extend previous findings of disorder-specific classification in adults with ASD, where the classifier was not suitable to classify adult patients with ADHD [54].

The pattern of brain regions that discriminated the ASD from the ADHD group is largely in areas that have been found to be different in ASD patients relative to controls such as in cerebellar hemispheres and cerebellar vermis, ACC caudate/thalamus, inferior frontal cortex, middle and superior temporal and right inferior parietal regions [55]–[58]. The discriminating brain regions identified for ASD have also been implicated in the mediation of three core behaviors that are impaired in ASD; namely social impairment (ACC, fusiform gyrus, inferior frontal cortex, and posterior parietal cortex), communication deficits (SMA, basal ganglia, substantia nigra, and thalamus), and repetitive behaviors (ACC, basal ganglia and thalamus) [59]. Furthermore, the finding of inferior parietal patterns classifying ASD versus ADHD echoes and extends the univariate VBM findings of the only structural MRI study which compared between ADHD and ASD adolescents and found ASD-specific enhanced GM relative to controls in right supramarginal gyrus [60]. The positive contribution for classifying ASD in the cerebellum is interesting in view of the only fMRI comparison between the two disorders during a vigilance task, where we showed that ASD patients had a disorder-specific cerebellar overactivation relative to both healthy controls and ADHD adolescents [61]. Finally, the disorder-specificity of the classifier is further confirmed by our 3-class classification that showed a far higher than chance accuracy of 75.9%, 65.5%, and 63.2%, in classifying ADHD, healthy controls and ADHD, by clearly distinctive structural GM patterns.

Together, the findings suggest that it is possible to use multivariate pattern recognition analyses for disorder-specific classification of ADHD based on structural imaging data. If replicated this may have future implications as a possible aid in differential diagnosis, in particular for difficult to diagnose patients.

The strength of the study is the use of probabilistic GPC methods that confers multiple benefits for clinical studies. Another strength is the inclusion of mostly (73%) medication-naïve ADHD patients, since long-term stimulant medication has been associated with more normal GM and cortical thickness of fronto-cingulate, parietal, cerebellar and striatal regions[2], [52], [62]–[65] and 100% medication-naïve ASD patients. To increase the homogeneity of the sample, we included only males with the combined hyperactive-impulsive and inattentive subtype of ADHD. However, this limits the diagnostic classification patterns to the male ADHD subtype. Also, future diagnostic utility of pattern classification analyses will rely on its ability to sub-classify even more refined ADHD subtypes such as attention deficit alone without hyperactivity or ADHD with emotional dysregulation based on their brain structure patterns.

A limitation is a relatively modest sample size. While the study advances neuroimaging towards providing useful diagnostic markers for ADHD, it does not definitively quantify their discriminative value. Also, patients differed in IQ, which is typical for this population [40]. However, no correlation was observed between IQ and GPC probabilities making it unlikely that IQ played a crucial factor. Lastly, while the overall classification accuracy of 79.3% and in particular the specificity of 81.5% was respectable, this would still leave a relatively large percentage of incorrectly classified individuals. Future studies in larger samples, perhaps including other structural measures such as cortical thickness, may provide better classification accuracy.

In conclusion, this is the first study demonstrating probabilistic GPC methods for accurately classifying ADHD patients based on their brain structure patterns. We achieved a considerable overall classification accuracy of 79.3% based on distributed and clearly distinct GM brain structure patterns, with later developing dorsolateral fronto-striato-parieto-cerebellar networks discriminating controls and earlier developing ventrolateral/premotor fronto-limbic–brain stem networks discriminating ADHD. Importantly, the classifier was both sensitive and specific for ADHD and was also disorder-specific relative to ASD. The findings are a promising step towards finding an objective differential diagnostic tool based on brain imaging measures to aid with the subjective clinical diagnosis of ADHD.

## Supporting Information

Figure S1 **Non-thresholded three-class** **multivariate discrimination weight maps.** A. Multivariate discrimination weight map for ADHD (orange) vs. Controls and ASD (light blue). B. Multivariate discrimination weight map for Controls (orange) vs. ADHD and ASD (light blue). C) Multivariate discrimination weight map for ASD (orange) vs. ADHD and Controls (light blue). The intensity values of the multivariate discrimination weight-maps illustrate the relative positive weight distributions (orange) and negative weight distributions (cyan).
(TIF)
