# Supplementary figures and images for: Disorder-Specific Predictive Classification of Adolescents with Attention Deficit Hyperactivity Disorder (ADHD) Relative to Autism Using Structural Magnetic Resonance Imaging
Source: PLoS One. 2013 May 16;8(5):e63660. doi: 10.1371/journal.pone.0063660 (PMC3656087; doi:10.1371/journal.pone.0063660)

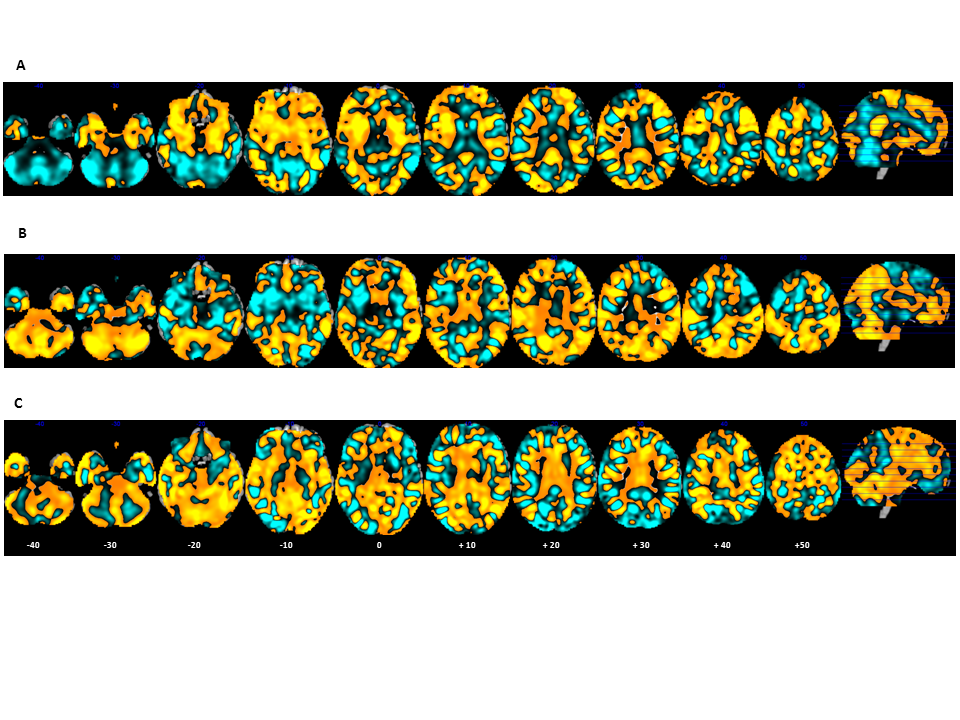

Supplement: Figure S1 — Non-thresholded three-class multivariate discrimination weight maps. A. Multivariate discrimination weight map for ADHD (orange) vs. Controls and ASD (light blue). B. Multivariate discrimination weight map for Controls (orange) vs. ADHD and ASD (light blue). C) Multivariate discrimination weight map for ASD (orange) vs. ADHD and Controls (light blue). The intensity values of the multivariate discrimination weight-maps illustrate the relative positive weight distributions (orange) and negative weight distributions (cyan). (TIF) [file pone.0063660.s001.tif]
